# Correction: Novel azobenzene-based amphiphilic copolymers: synthesis, self-assembly behavior and multiple-stimuli-responsive properties

**DOI:** 10.1039/c8ra90036a

**Published:** 2018-05-16

**Authors:** Yiting Xu, Jie Cao, Qi Li, Jilu Li, Kaiwei He, Tong Shen, Xinyu Liu, Conghui Yuan, Birong Zeng, Lizong Dai

**Affiliations:** Fujian Provincial Key Laboratory of Fire Retardant Materials, Xiamen University Xiamen 361005 People’s Republic of China xyting@xmu.edu.cn +86 18750256597

## Abstract

Correction for ‘Novel azobenzene-based amphiphilic copolymers: synthesis, self-assembly behavior and multiple-stimuli-responsive properties’ by Yiting Xu *et al.*, *RSC Adv.*, 2018, **8**, 16103–16113.

The final entry for Table 1 relating to Sample C-4 in the published paper showed an incorrect entry for the column [POSSMA] : [AZOMA] : [DMAEMA] : [CDB] : [AIBN]; this should read 12 : 50 : 100 : 0 : 0.20.

The Royal Society of Chemistry apologises for these errors and any consequent inconvenience to authors and readers.

## Supplementary Material

